# Identification of ciguatoxins in a shark involved in a fatal food poisoning in the Indian Ocean

**DOI:** 10.1038/s41598-017-08682-8

**Published:** 2017-08-15

**Authors:** Jorge Diogène, Laia Reverté, Maria Rambla-Alegre, Vanessa del Río, Pablo de la Iglesia, Mònica Campàs, Oscar Palacios, Cintia Flores, Josep Caixach, Christian Ralijaona, Iony Razanajatovo, Agathe Pirog, Hélène Magalon, Nathalie Arnich, Jean Turquet

**Affiliations:** 1 Marine Environmental Monitoring, IRTA, Ctra. Poble Nou, km 5.5, 43540 Sant Carles de la Ràpita, Spain; 2 Mass Spectrometry Laboratory/Organic Pollutants, IDAEA-CSIC, Jordi Girona 18, 08034 Barcelona, Spain; 3 IHSM, Institut Halieutique des Sciences Marines de Tuléar, Université de Toliara, Toliara, Madagascar; 4 IPM Institut Pasteur Madagascar, Laboratoire d’Epidémio-Surveillance, BP 1274 - Avaradoha, 101, Antananarivo, Madagascar; 5 UMR ENTROPIE Univ. Réunion/IRD/CNRS, Faculté des Sciences et Technologies, Université de La Réunion, 15 Bd René Cassin, CS 92003, 97744 St Denis Cedex 09, La Réunion, France; 6 ANSES French Agency for Food, Environmental and Occupational Health & Safety, Unit on Food Risk Assessment, Risk Assessment Department, 14 rue Pierre et Marie Curie - 94701, Maisons-Alfort Cedex, France; 7 HYDROREUNION, CBEM, C/O CYROI, 2, Rue Maxime Rivière, 97490 Sainte Clotilde, La Réunion France

## Abstract

Severe food poisoning events after the consumption of sharks have been reported since the 1940s; however, there has been no clear understanding of their cause. Herein, we report for the first time the presence of ciguatoxins (CTXs) in sharks. The identification by mass spectrometry of CTXs, including two new analogues, in a bull shark (*Carcharhinus leucas*) that was consumed by humans, causing the poisoning and death of 11 people in Madagascar in 2013 is described. Typical neurotoxic ciguatera symptoms were recorded in patients, and toxicological assays on extracts of the shark demonstrated CTX-like activity. These results confirm this episode as a ciguatera poisoning event and expand the range of pelagic fish species that are involved in ciguatera in the Indian Ocean. Additionally, gambieric acid D, a molecule originally described in CTX-producing microalgae, was identified for the first time in fish. This finding can contribute to a better understanding of trophic relations within food webs. The present work confirms that consumption of sharks from the Indian Ocean should be considered a ciguatera risk, and actions should be taken to evaluate its magnitude and risk in order to manage shark fisheries.

## Introduction

Ciguatera is a well-known food poisoning that occurs when fish containing ciguatoxins (CTXs) are consumed. These potent neurotoxins are produced by microalgae of the genus *Gambierdiscus* ^1–3^ and *Fukuyoa* ^4, 5^. Ciguatoxins produced by these microalgae may be transferred along the food web, eventually reaching carnivorous fish like barracuda or amberjack. Ciguatera is the main cause of seafood poisoning due to the consumption of fish, and estimations point out around 50,000–500,000 people are affected by ciguatera each year^6^, although these should be re-evaluated for a better assessment of the present impact of ciguatera.

Numerous incidences of human poisoning after the consumption of several species of shark have been reported since the 1940s. These cases have been proposed to be ciguatera events according to the toxicity in animal assays or due to the symptoms in patients^7^. However, the presence of CTXs has never been confirmed in sharks. In Madagascar, a first possible event of ciguatera was described in 1993, after the consumption of shark in Manakara (south-east coast) and was noted for its unprecedented severity. Several hundred people (between 200 and 500 depending on the different authors) were poisoned due to the consumption of a shark, either a bull shark (*Carcharhinus leucas*) or a pigeye shark (*C. amboinensis*), two species that are difficult to distinguish. This event resulted in the death of between 60 and 98 people, depending on the different authors, a fatality rate of 20 to 30%^8, 9^. In this particular event, patients presented almost exclusively neurological symptoms. Boisier *et al*. identified two toxic extracts from the liver of the shark, which were proposed to be the causative agent of the poisoning, and tentatively named the new toxins as carchatoxin-A and carchatoxin-B^9^. However, the toxicity levels of the shark flesh did not match that of the liver extracts, and thus, toxicity remained unexplained. No further information regarding the chemical behaviour, structure, toxicity or mechanism of action of carchatoxins has been published since then. Another event, occurring between November 14 and 19, 2013 at Fenerive-Est in Madagascar, caused the poisoning of 97 people that presented ciguatera symptoms after eating the flesh, the liver or the head of a bull shark, 11 of whom died. Our preliminary laboratory results of this particular event were communicated to the French “Agence nationale de sécurité sanitaire de l’alimentation, de l’environnement et du travail” (ANSES) in order to quickly manage the potential risk of food poisoning by shark consumption in the Madagascar area^10^. Further data regarding the epidemiology of this event, described that the major symptoms were neurological and digestive^11^.

We report herein the confirmation of ciguatera, caused by consumption of this bull shark (*C. leucas*) in Madagascar in November 2013. This was based on the evidence of symptoms in patients and in mice, cellular toxicity, and unequivocal identification of CTXs by liquid chromatography coupled to high resolution mass spectrometry (LC-ESI-HRMS). To the best of our knowledge, this is the first identification of CTXs in sharks.

## Results

### Poisoning event in Madagascar

**Figure 1:**
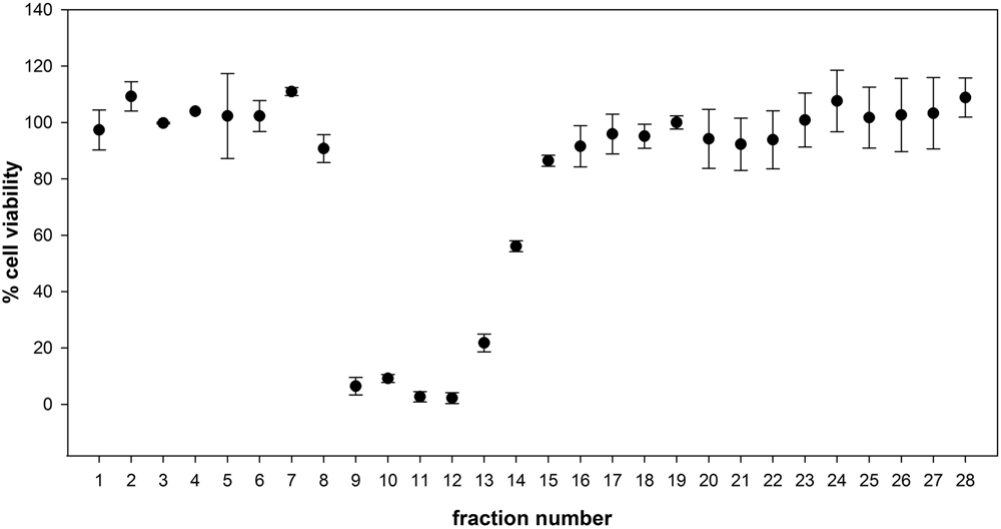
Toxicity of stomach fractions by cell-based assay (CBA). Toxicity was estimated according to the cell viability obtained after exposing cells to 0.27 µL of each fraction/mL. Error bars represent standard deviation (SD) values for 3 replicates (n = 3).

In November 2013, an outbreak of fish poisoning following the consumption of shark was reported in the district of Fenoarivo Atsinanana (Fenerive-Est, Madagascar). According to the information transmitted to the ANSES by the “French Institute for Public Health Surveillance,” dated 22 April 2014, 124 people, 11 of whom died, were poisoned after consuming the flesh, liver, head and part of the viscera of a shark^11^. The patients developed symptoms between 2 and 12 h following ingestion of their meal, and the predominant neurological signs were paraesthesia of the extremities, dysesthesia, and reversing sensitivity of hot and cold. These symptoms were accompanied by headache, dizziness, and arthralgia. The digestive symptoms were moderate and inconsistent. The clinical profile was similar to that of patients that had previously been poisoned after consumption of shark in Madagascar^9^. A detailed epidemiological report is presented by Rabenjarison *et al*.^11^, and additional investigations conducted by agents of the “Institut Halieutique et des Sciences Marines” (Tulear, Madagascar) concluded that the shark in question was a female of about 1.5 m in length. Samples of the fish implicated in the episode, and used in our study, consisted of salted stomach, three dried fins, and partially cooked flesh. The genetic analyses that we performed using 22 microsatellite loci on the five samples demonstrated that they all belong to the species *C. leucas* and surely to the same individual (identical multilocus genotypes; Supplementary Table S1 and Fig. 1).

### Toxicity evaluation of shark samples by mouse bioassay (MBA)

The MBA performed on the flesh samples showed toxicity, although quantification was not possible due to the limited amount of samples. The single dose injected corresponds to an amount of 50 g equiv. flesh/mouse. The symptoms observed in the mice included: paralysis of limbs, dyspnoea, convulsions, mild diarrhea, and mortality from respiratory failure between 3 and 4 hours after injection of the extract. Toxicity and symptoms in mice were similar to those previously described in the study on shark toxins in Madagascar^9^. The MBA performed on the stomach sample showed a very high toxicity. Even at low doses, when mortalities were observed these were always rapid (survival time less than 1.5 h) and otherwise the mice recovered. The symptoms observed were dominated by neurological problems, including difficulty with breathing, followed by a severe respiratory arrest. In some mice, hyper-salivation was also observed. The lowest dose tested that resulted in the death of mice was estimated at 72 mg equiv. stomach per mouse of 20–22 g. Fin samples were not tested by MBA.

### Evaluation of ciguatoxin-like activity by Neuro-2a cell-based assay (CBA)

Neuro-2a cells exposed to Pacific ciguatoxin P-CTX-1 (CTX1B) standard presented the expected dose-response curve with a regression factor (*R* ^2^) of 0.996. The concentration of P-CTX-1 that caused a 50% cell mortality (IC_50_) was 19.9 pg/mL. The limit of detection (LOD), defined as the concentration of P-CTX-1 that causes a 20% of cell mortality (IC_20_), was 8.4 pg/mL (Supplementary Figure S2). Considering a maximum exposure concentration of 200 mg/mL for flesh, 100 mg/mL for stomach and fins 1 and 3, and 50 mg/mL for fin 2 (Supplementary Figure S3), the effective LODs (eLODs) for P-CTX-1 in shark samples were 0.04 µg P-CTX-1 equiv./kg for flesh, 0.08 µg P-CTX-1 equiv./kg for stomach and fins 1 and 3, and 0.17 µg P-CTX-1 equiv./kg for fin 2. Flesh, stomach and fins 1 to 3 crude extracts of *C. leucas* contained 0.06, 92.78, 0.12, 0.79 and 0.17 µg P-CTX-1 equiv./kg matrix, respectively (Table 1).

**Table 1:** Concentration of P-CTX-1 equiv./kg tissue in crude stomach, flesh and fin extracts as determined by mouse bio-assay (MBA), Neuro-2a cell-based assay (CBA) and liquid chromatography coupled to high resolution mass spectrometry (LC-ESI-HRMS).

Crude extract	MBA (µg P-CTX-1 equiv./kg tissue)	CBA (µg P-CTX-1 equiv./kg tissue)	LC-ESI-HRMS (µg P-CTX-1 equiv./kg tissue)		
			I-CTX-1&2	I-CTX-3&4	Σ I-CTXs
flesh	n.q.	0.06	n.d.	n.d.	n.d.
stomach	83	92.78	6.54	9.74	16.28
fin 1	—	0.12	—	—	—
fin 2	—	0.79	n.d.	n.d.	n.d.
fin 3	—	0.17	—	—	—

With the aim of separating the different compounds to better identify the toxin profile of the most toxic sample, the stomach extract was fractionated by high-performance liquid chromatography (HPLC). In order to identify the distribution of the toxin within the 28 fractions recovered, cells were first exposed to 2.17 µL of each fraction/mL. CTX-like activity was observed in fractions F8 to F22. Fractions F8 and F17 to F22 fell within the working range (IC_20_-IC_80_), and CTX-like content was able to be quantified. The use of lower fraction volume (0.27 µL of each fraction/mL) was required to quantify fractions F13 and F14 (Fig. 1). Further dilution was required to quantify fractions F9 to F12. Distribution of ciguatoxins after fractionation of the stomach crude extract is shown in Table 2.

**Table 2:** Distribution of ciguatoxins (CTXs) after fractionation of the stomach crude extract. Percentages of P-CTX-1 equiv. recovered in each fraction in relation to the P-CTX-1 equiv. injected, estimated by the Neuro-2a cell-based assay (CBA) and liquid chromatography coupled to high resolution mass spectrometry (LC-ESI-HRMS).

Fractions	CBA (% P-CTX-1 equiv.)	LC-ESI-HRMS (% P-CTX-1 equiv.)		
		I-CTX-1&2	I-CTX-3&4	Σ I-CTXs
F8	0.23	n.d.	n.d.	n.d.
F9	5.15	n.d.	5.67	5.67
F10	5.80	n.d.	29.66	29.66
F11	9.37	13.87	15.75	29.62
F12	8.05	16.12	3.86	19.98
F13	1.79	n.d.	n.d.	n.d.
F14	1.44	n.d.	n.d.	n.d.
F15	2.30	n.d.	n.d.	n.d.
F16	0.62	n.d.	n.d.	n.d.
F17	0.29	n.d.	n.d.	n.d.
F18	0.27	n.d.	n.d.	n.d.
F19	0.33	n.d.	n.d.	n.d.
F20	0.23	n.d.	n.d.	n.d.
F21	0.19	n.d.	n.d.	n.d.
F22	0.12	n.d.	n.d.	n.d.

### Confirmation of ciguatoxins (CTXs) by liquid chromatography coupled to high resolution mass spectrometry (LC-ESI-HRMS)

A liquid chromatography electrospray ionization high-resolution mass spectrometry (LC-ESI-HRMS) method was developed for the analysis of CTXs in extracts of *C. leucas*, based on previous LC-MS/MS methods^3, 12, 13^. The spectra of I-CTXs were dominated by [M+H]^+^, [M+NH_4_]^+^, [M+Na]^+^, [M+H-H_2_O]^+^, [M+H-2H_2_O]^+^ in accordance with Hamilton *et al*.^13^. The adduct ions giving higher signals, specifically [M+NH_4_]^+^ and [M+Na]^+^, were chosen for confirmation and quantification purposes.

Crude extracts of flesh, stomach and fin 2 were analyzed by LC-ESI-HRMS. The presence of CTX analogues was not observed in the flesh nor in the fin 2 crude extracts. A possible explanation is that the LOD attained by CBA (0.04 µg P-CTX-1 equiv./kg flesh tissue and 0.17 µg P-CTX-1 equiv./kg fin 2 tissue) is lower than the LOD attained by LC-ESI-HRMS (0.5 µg P-CTX-1 equiv./kg tissue). In the stomach crude extract, the CTX analogues I-CTX-1&2 and I-CTX-3&4 were detected and quantified (Table 1).

All analogues were confirmed using their theoretical accurate mass (*m/z*), measured *m/z*, and mass accuracy (ppm): i) I-CTX-1&2 ([C_62_H_92_O_19_NH_4_]^+^ and [C_62_H_92_O_19_Na]^+^): 1158.6571, 1158.6606, <5.49 ppm and 1163.6125, 1163.6148, <4.93 ppm, respectively; and ii) I-CTX-3&4 ([C_62_H_92_O_20_NH_4_]^+^ and [C_62_H_92_O_20_Na]^+^): 1174.6540, 1174.6565, <6.31 ppm and 1179.6084, 1179.6146, <5.89 ppm, respectively. From a qualitative point of view, both LC-ESI-HRMS and CBA showed the presence of P-CTX-1 equiv. in the stomach crude extract. However, lower contents of P-CTX-1 equiv. were estimated by LC-ESI-HRMS in relation to the CBA. This difference in the quantification could be attributed to the different principles of the techniques: while LC-ESI-HRMS is based on structural identification of specific CTX analogues, and may neglect some non-described CTX analogues, CBA measures a composite toxicity, which is a global response indicative of the toxic effect of several CTX analogues on cells.

**Figure 2:**
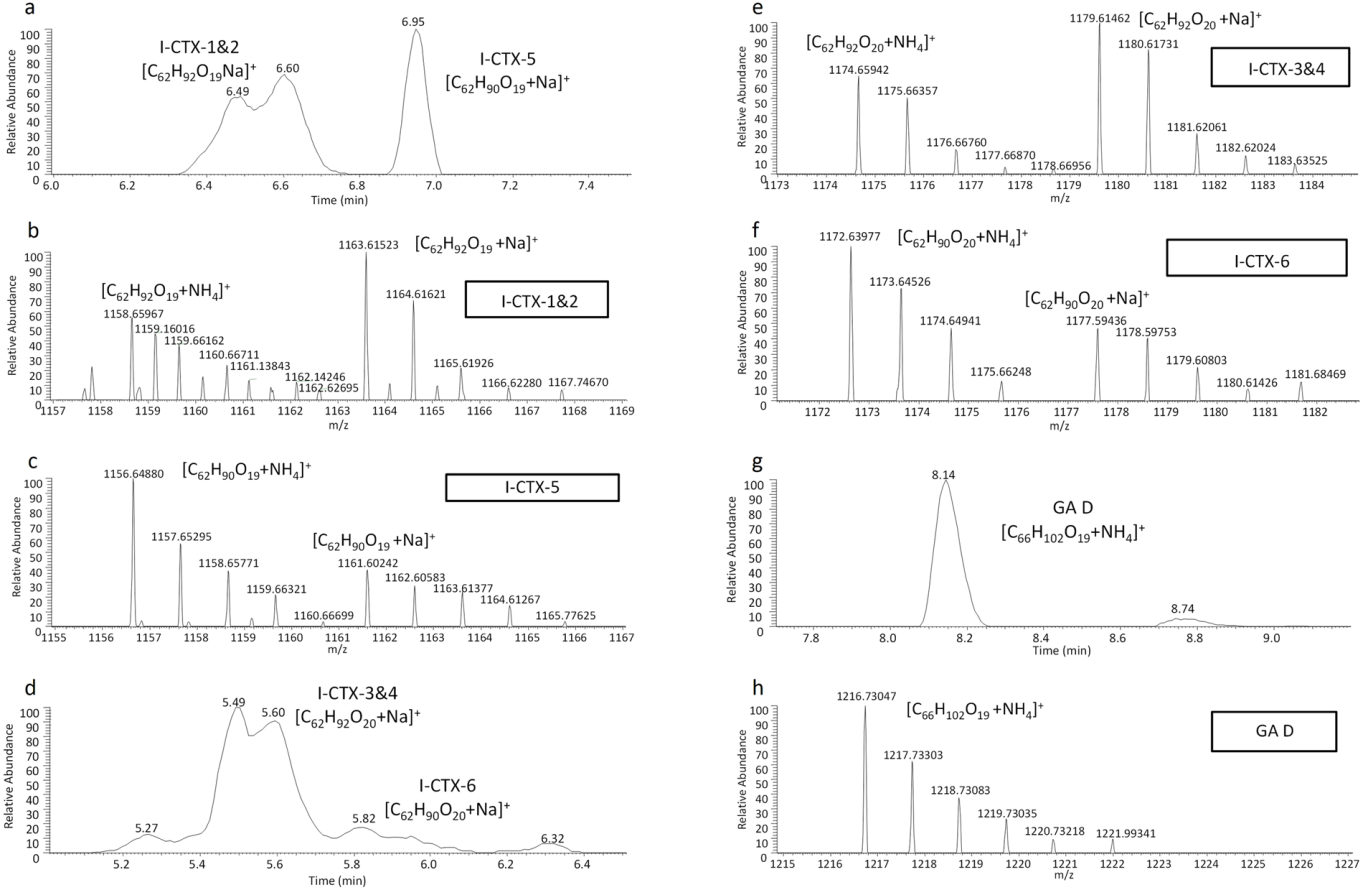
Evidence for the presence of of ciguatoxins (CTXs) and gambieric acid D (GA D) in shark tissues. (**a**) Extracted ion chromatogram of I-CTX-1&2 [M+Na]^+^ at 1163.6125 and HRMS exact mass spectra of (**b**) I-CTX-1&2 [M+Na]^+^ at 1163.6125 and (**c**) I-CTX-5 (C_62_H_90_O_19_) [M+Na]^+^ at 1161.6070, in fraction F12 from stomach; (**d**) extracted ion chromatogram of I-CTX-3&4 ([M+Na]^+^ at 1179.6084 and HRMS exact mass spectra of (**e**) I-CTX-3&4 [M+Na]^+^ at 1179.6084 and (**f**) I-CTX-6 (C_62_H_90_O_20_) [M+Na]^+^ at 1177.5910, in stomach crude; (**g**) extracted ion chromatogram of GA D [M+NH_4_]^+^ at 1216.7354 and (**h**) HRMS exact mass spectra of GA D [M+NH_4_]^+^ at 1216.7354, in flesh crude extract, respectively.

Having identified the toxic stomach fractions using the CBA, fractions F8 to F22 were analyzed by LC-ESI-HRMS for toxin identification. Fractionation of the stomach crude extract reduced matrix interferences and confirmed the presence of I-CTX-1&2 and/or I-CTX-3&4 in fractions F9 to F12, the most toxic ones by CBA (Table 2). Extracted ion chromatograms for I-CTX-1&2 and I-CTX-3&4 found in fraction F12 from stomach are shown in Fig. 2a and d, respectively. Full HRMS exact mass spectra of I-CTX-1&2 and I-CTX-3&4 (Fig. 2b and e, respectively) confirmed the presence of these toxins. The isotopic pattern of each signal was taken into consideration in assigning their molecular formula. In addition, these toxins showed a profile similar to P-CTX-1 according to [M+NH_4_]^+^ and [M+Na]^+^. As for the analysis of crude extracts, the percentages of P-CTX-1 equiv. in fractions F9 to F12 determined by LC-ESI-HRMS were higher than those obtained by CBA. Nevertheless, both techniques concluded that fractions F9 to F12 contained the highest CTX content among all fractions.

Two new CTX analogues (I-CTX-5 and I-CTX-6) were detected by LC-ESI-HRMS (Fig. 2c and f). The identification of these new CTX analogues related to I-CTXs in the stomach crude extract and fractions was given using the restrictive criteria to propose elemental formulae. Ion assignment indicated that the new CTX analogues had nearly the same molecular formula as I-CTX-1&2 and I-CTX-3&4 with only 2H less, which corresponds to the formation of a double bond. The ring double bond equivalents (RDBEs) for both unknown compounds was 16.5, which corresponds to the 15.5 RDBE value from CTX analogues (I-CTX-1&2 and I-CTX3&4). Analogues were confirmed according to their theoretical accurate *m/z*, measured *m/z*, and mass accuracy (ppm): i) I-CTX-5 ([C_62_H_90_O_19_NH_4_]^+^ and [C_62_H_90_O_19_Na]^+^): 1156.6414, 1156.6479, 5.57 ppm and 1161.5968, 1161.6030, 5.29 ppm, respectively; and ii) I-CTX-6 ([C_62_H_90_O_20_NH_4_]^+^ and [C_62_H_90_O_20_Na]^+^): 1172.6496, 1172.6381, −1.44 ppm and 1177.5918, 1177.5953, 3.03 ppm in fraction F12 from stomach, respectively. Full HRMS exact mass spectra of both new I-CTXs in Fig. 2c and f confirm the presence of these toxins.

### Identification of gambieric acid D (GA D) by liquid chromatography coupled to high resolution mass spectrometry (LC-ESI-HRMS)

Gambieric acid D (C_66_H_102_O_19_) (GA D), which is also produced by *Gambierdiscus* spp., was identified in the flesh crude extract of the shark using the theoretical accurate *m/z*, measured *m/z*, and mass accuracy (ppm) of [C_66_H_102_O_19_NH_4_]^+^: 1216.7354, 1216.7304, −4.02 ppm at 8.14 min, respectively. Quantification by LC-ESI-HRMS was not carried out due to the lack of GA D standard solutions. Extracted ion chromatograms (Fig. 2g) and HRMS exact mass spectra (Fig. 2h) confirm the presence of GA D by the *m/z* of most abundant ion peaks [M+NH_4_]^+^. No GA D was identified in the stomach or in the fins.

## Discussion

The methodology presented in this paper, which combines a multi-disciplinary approach focused on epidemiology, toxicology and instrumental analysis, has proved to be effective in the identification of CTXs in seafood and contributes to a better characterization of the present incidence of ciguatera.

This is the first evidence of the presence of CTXs in sharks. The identification of CTXs in the shark responsible for a food poisoning event in Madagascar presented herein, along with additional observations, confirm the episode as a ciguatera event and the suspected implication of sharks in ciguatera^7^. The evidences that support this confirmation are: i) the symptoms observed in patients matched those of ciguatera, ii) injection of flesh and stomach crude extracts to mice resulted in symptoms characteristic of CTXs, which were comparable to those reported for I-CTX in the bony fish *Lutjanus sebae* from the Indian Ocean^13^, iii) neuroblastoma cells exposed to flesh, stomach and fin crude extracts showed the characteristic toxicity of CTXs through activation of voltage-gated sodium channels, and iv) LC-ESI-HRMS provided the identification of I-CTX- 1&2 and I-CTX-3&4 in the stomach extracts. The stomach was extremely toxic with an estimation of 92.78 µg P-CTX-1 equiv./kg by cell-based assay (CBA), a concentration approximately 10,000 times the guidance level concentration of P-CTX-1 causing ciguatera in humans, established at 0.01 µg P-CTX-1/kg by the FDA^14^, and considered by EFSA^15^ as the level expected not to exert effects in sensitive individuals. The estimation of the stomach levels by LC-ESI-HRMS was lower than determined by CBA, 16.28 P-CTX-1 equiv./kg; however, this level is well above the FDA guidance level. Flesh and fins presented toxicity with an estimation by CBA of 0.06 µg P-CTX-1 equiv./kg in the flesh and 0.12, 0.79 and 0.17 µg P-CTX-1 equiv./kg in fins 1 to 3, respectively, which are also above the FDA guidance level. Identifying CTXs in viscera is significant, since local food habits from the Indian Ocean include eating liver and viscera^11^, some of which are dried and salted. Since the liver of the shark was not available, the possible presence of carchatoxins previously described in other shark poisoning events^16^ could not be studied.

Currently, only four I-CTX analogues (I-CTX-1&2 and I-CTX-3&4) have been described in the literature^13^. As for the already known CTXs, I-CTX-1&2 and I-CTX -3&4, our results obtained in the stomach of shark revealed higher amount of I-CTX-3&4 in relation to I-CTX-1&2 (60% vs 40% of the total amount of I-CTXs estimated by LC-ESI-HRMS). Contrarily, in that previous study on Indian CTXs in one fish (*Lutjanus sebae*), a lower amount of I-CTX-3&4 in relation to I-CTX-1&2 was described^13^. This difference may be explained by the tissue evaluated, since in their work the whole fish was analysed^13^. Additionally, Hamilton and collaborators postulated that I-CTXs-1&2 might originate from dinoflagellates and that I-CTX-3&4 would be metabolites produced in fish^13^. Since sharks are higher in the trophic webs than *L. sebae*, this may explain the higher amounts of I-CTX3&4 in relation to I-CTX-1&2 obtained in shark stomach. Herein, two new I-CTX analogues have been identified by LC-ESI-HRMS in the stomach extract and fractions, I-CTX-5 showing 2H less than I-CTX-1&2 and I-CTX-6 showing 2H less than I-CTX-3&4, which corresponds to the formation of a double bond. This result widens the number of CTXs analogues possibly present in nature^3, 12, 13^ and this will impact our understanding of ciguatera. First, the identification of new CTX analogues may indicate that the metabolism of CTXs may be more complex than previously foreseen. Second, these new CTX analogues possibly present in seafood will need to be taken into account for a better evaluation of ciguatera risks.

Gambieric acid D (GA D)^17^ was identified in shark flesh. To the best of our knowledge, this work is the first report of GA in any organism other than *Gambierdiscus* spp.^16^. The identification of GA D in the flesh of the shark evidences, additionally to the presence of CTXs, the link between *Gambierdiscus* spp. and this particular food poisoning event. Identifying GA D in sharks that, as carnivorous pelagic fish are situated at the highest levels of the marine food webs, demonstrates how stable these molecules may be throughout their transfer and metabolic transformations along the food webs. The identification of molecules produced by microorganisms in animals situated at higher trophic levels, such as GA D would constitute, as for the analysis of fatty acids or stable isotopes, a good strategy to understand trophic relations in the ecosystems. Gambieric acid A has demonstrated no toxicity in mice, while a mixture of GA C and GA D was moderately toxic to mouse lymphoma cells L5178Y^18, 19^. Additionally, GA A has been demonstrated to bind to the voltage gated sodium channels in synaptosomes isolated from rat brains^20^ in the same manner as CTXs, but with much less affinity to these than CTXs, possibly explaining its low toxicity. To better understand the potential harmful effects of GAs, and more specifically of GA D, their toxicity should be further characterized; however, at this moment reference material for GAs is not commercially available. Urgent need exists for the availability of certified standards for CTXs and GAs.

About 100 million sharks are caught each year world-wide^21^, and the global shark fin trade is estimated to be worth US $ 400–500 million a year^22^. The identification of CTXs in shark from the Indian Ocean may favour the re-consideration of local food safety measures that could affect the shark fisheries industry, which is of special relevance in areas such Madagascar^22^. The present work confirms that shark consumption, in this example, a bull shark (*C. leucas*), from the Indian Ocean should be considered a ciguatera risk, and actions should be taken to evaluate its magnitude and risk in order to manage shark fisheries. As for the numerous suspicious cases of ciguatera involving sharks^7^, it may be postulated that sharks with CTXs will not be restricted to the species *C. leucas* and to the Indian Ocean. Consequently, other species of shark and other oceans should also be considered for ciguatera evaluation, especially to account for the migration of sharks and current changes in the geographical distribution of sharks due to fishing pressure and global warming^23, 24^.

## Methods

### Samples

Shark samples were collected and analyzed in the framework of a research and development agreement funded by ANSES. Five samples were recovered by the health authorities of Madagascar “Agence de Contrôle Sanitaire et de la Qualité des Denrées Alimentaires de Madagascar” (ACSQDA) and were transferred to the laboratory by the WHO and the “Pasteur Institute of Madagascar”. The salted stomach, three dried fins and partially cooked flesh samples were stored at −25 °C until extraction. Samples of flesh and stomach were crushed and homogenized before starting the analyses. Until extraction, they were stored at −25 °C.

### Shark species identification through microsatellite genotyping and Dloop sequencing

The five samples (flesh, stomach and fins 1 to 3) from elasmobranch tissues, suspected to be from bull shark (*C. leucas*), were used for identification. Total genomic DNA was extracted using Qiagen DNeasy Blood & Tissue kit (Qiagen, Hilden, Germany). The mitochondrial control region (D-loop; 832 bp) was also sequenced using primers designed previously^25^, but failed to amplify due to DNA degradation before collection. Therefore, genotyping was performed using a total of 22 microsatellite loci [20 loci developed for *C. leucas* ^26^, one locus (Cli106) for *C. Limbatus* ^27^ and one locus (Gc01) for *Galeocerdo cuvier* ^28^]. All amplifications were performed as described in the literature^26^. Then, identical multi-locus genotypes (MLGs) were identified using the software GenClone v. 2.0^29^ and, using the software STRUCTURE v. 2.3.1^30^, assignment tests were performed together with MLGs obtained from other carcharhinid shark species (*C. leucas, C. obscurus* and *C. plumbeus*) that are known to amplify with the same loci.

### Toxin standards

Pacific type 1 CTX (P-CTX-1 or P-CTX-1), P-CTX-2 and P-CTX-3 standard solutions were provided by Pr. Richard J. Lewis (The Queensland University, Australia). P-CTX-1 standard was used for CBA and LC-ESI-HRMS analysis. P-CTX-2, P-CTX-3 standards were used only for LC-ESI-HRMS analysis.

### Flesh, stomach and fins extraction

Samples were extracted and purified according to the protocol described in the literature^31^ with minor modifications provided by ANSES. In brief, 10 g ± 0.1 g of flesh, stomach or fins homogenates were placed in 50-mL tubes. Samples were extracted in 20 mL of acetone and homogenized with an Ultraturrax blender. Samples were heated in the sealed tube at 70 °C for 10 min in a water bath. The supernatant was recovered by centrifugation at 3,000 g for 10 min at 4 °C and filtered using 0.45-µm nylon filters. The sample pellets were re-extracted with acetone and supernatants were pooled and evaporated until dry. Liquid/liquid partition was then performed twice in the tubes with 20 mL of water/diethyl ether (DEE) (1:4, v-v). The DEE upper phase was recovered and pooled with the second DEE partition. Both DEE phases were evaporated until dry. The dried extracts were then dissolved in 4 mL of *n*-hexane and 2 mL of methanol/water (4:1, v-v). The hexane upper phase was removed. This liquid/liquid partition was repeated three times and the methanol phases were pooled and evaporated until dry. Finally, the resulting residues were re-dissolved in 4 mL of HPLC-grade methanol and preserved at −20 °C until analyzed.

### Stomach extract fractionation

A total of 2,750 µL of stomach extract were evaporated until dry using N_2(g)_, and re-dissolved in 1,000 µL of HPLC-grade methanol. The analytical fractionation of this extract was performed as described before for the chromatographic separation of CTXs^3^. Once the chromatographic run started, fractions were collected every 30 seconds (n = 28). After fractionation, the volume of each tube was evaporated to dryness, re-dissolved in 500 µL of HPLC-grade methanol and preserved at −20 °C until analyzed. Stomach fractions were analyzed by LC-ESI-HRMS and CBA, but not by MBA.

### Mouse bioassay

The protocol used at HYDROREUNION was validated beforehand by the ethics committee (Protocol agreement n° EU0450 - GIP CYROI - APAFiS - Autor. APAFiS #2641-2015110916009490) and was in accordance with the regulations in force. This protocol is based on a standard method developed by ANSES (CATNAT-10). The extracts of shark were solubilized in Tween-60 1–5% saline solution, and then injected into three mice (male, OF1; 20 ± 2 g) by intraperitoneal (i.p.) route. The mice were observed continuously during the first 2 h, and then monitored regularly up to 24 h after injection. The interpretation of the results was based on the symptoms observed and the time-to-death of the mice. The typical symptoms of the presence of CTXs include profuse diarrhea, piloerection, respiratory disorders, dyspnoea and, when using male mice, transient pre-erectional cyanosis of the penis (which can become priapism). With the Indian Ocean toxins, this last symptom is observed only very rarely. It therefore does not appear in the classical description for this region. The death of 1 or 2 mice within 24 h was deemed a positive result indicating the presence of CTXs (sample therefore non-edible).

### Assessment of ciguatoxin-like activity by Neuro-2a cell-based assay

Neuroblastoma mice (Neuro-2a cell line: CCL-131) were purchased from the American Type Culture Collection (ATCC) (LGC standards S.L.U., Barcelona, Spain). The presence of CTX-like activity in shark tissues extracts was evaluated on Neuro-2a cells according to the method based on the use of ouabain and veratridine published by Caillaud and co-workers^32^. Briefly, cells were exposed to the P-CTX-1 standard and to shark extracts for 24 h, and the CTX-like activity was measured in the presence of ouabain and veratridine with the MTT colorimetric assay^33^. Previous to the analysis of flesh, stomach and fins crude extracts or stomach fractions by CBA, methanol was removed from the extracts/fractions and P-CTX-1 standard solution by evaporation under N_2(g)_ and re-dissolved in RPMI medium. CTX-like activity was estimated with respect to P-CTX-1.

### Liquid chromatography coupled to high resolution mass spectrometry (LC-ESI-HRMS)

An Orbitrap-Exactive HCD (Thermo Fisher Scientific, Bremen, Germany) mass spectrometer equipped with heated electrospray source (H-ESI II), a Surveyor MS Plus pump and an Accela Open AS auto-sampler kept isothermal at 15 °C (Thermo Fisher Scientific, San Jose, California) were used for the analysis by LC-ESI-HRMS.

The chromatographic separation was performed on a reversed-phase Hypersil Gold C_18_ (50 mm × 2.1 mm, 1.9 µm) (Thermo Fisher, Scientific, Bremen, Germany) at a flow rate of 250 µL/min. Mobile phase A was water and B was acetonitrile/water (95:5), both containing 2 mM ammonium formate and 0.1% formic acid. The gradient elution program for the analysis was: 30% B 1 min, 30–40% B 2 min, 40–50% B 1 min, 50–90% B 5 min, 90% B 3 min and return to initial conditions for re-equilibrate (11 min 30% B). A 5-µL injection volume was used. The total duration of the method was 25 min.

The analyses were carried out in positive electrospray ionization (ESI+) mode, and the instrument was calibrated daily. P-CTX-1 was used to optimize the source, transmission and HRMS conditions in positive mode. The final parameters were: spray voltage of 4.0 kV, capillary temperature of 275 °C, heater temperature of 300 °C, sheath gas flow rate of 35 psi and auxiliary gas flow rate of 10 (arbitrary units). In addition, capillary voltage of 47.5 V, tube lens voltage of 186 V and skimmer voltage of 18 V were used. Nitrogen (purity > 99.999%) was employed as sheath gas, auxiliary gas and collision gas. The mass range was *m/z* 400–1,500 in full scan acquisition mode. The resolution was 50,000 (*m/z* 200, FWHM) at a scan rate 2 Hz, the automatic gain control (AGC) was set as “balanced” (1e^6^) with a maximum injection time of 250 ms. The data was processed with Xcalibur 2.2 SP1 software (Thermo Fisher Scientific, Bremen, Germany).

Automatic identification/quantification were performed. The peaks were extracted from the chromatogram using the exact mass of both [M+NH_4_]^+^ and [M+Na]^+^ diagnostic ions, the mass accuracy (±10 ppm extraction window), and the retention time window. In addition to HRMS and accuracy parameters for identification, in the present study, to be confident of the identification and the proposed elemental formulae, the following restrictive criteria were applied: elements considered were restricted in accordance with CTXs molecular formulae and adduct signals [C 55 to 70, H 64 to 110, O 11 to 25, N 0 to 1, and cations (Na) 0 to 1]; the isotopic pattern was matched and the charge, the ring double bond equivalents (RDBEs) and nitrogen rule were taken into account. Additionally, the monoisotopic pattern (M+1 ion) of these signals was used to assist in the further confirmation of the toxin’s identity. Therefore, in total four diagnostic signals were used for toxin identification. The relative ion intensities between [M+NH_4_]^+^, [M+Na]^+^ and their M+1 ions were calculated and matched taking into account a tolerance according to the EU Decision 2002/657/EC. The toxins in study are characterised by a vast amount of carbon atoms, leading to relative abundances of the ^13^C isotopic ion higher than 65%. This high sensitivity of M+1 ion render these signals a high identification potential. The combination of high resolution, AMM and restrictive criteria was crucial for identification of both targeted and unknown compounds, as well as precise quantification of analytes. An external standard calibration was carried out from 12.5 to 100 ng/mL using P-CTX-1 and showed good linearity (R^2^ = 0.996), with the LOD being 1.25 ng/mL. Due to the lack of proper analytical standards for all CTX congeners, in order to calculate concentrations of CTX analogues it was assumed that related analogues would give a similar response to that obtained with the P-CTX-1 standard.

## Electronic supplementary material

Supplementary information
