# Supplementary material for: Identification of ciguatoxins in a shark involved in a fatal food poisoning in the Indian Ocean
Source: Sci Rep. 2017 Aug 15;7:8240. doi: 10.1038/s41598-017-08682-8 (PMC5557899; doi:10.1038/s41598-017-08682-8)
Supplement: Supplementary file 1 — Supplementary information [file 41598_2017_8682_MOESM1_ESM.pdf]

Identification of ciguatoxins in a shark involved in a fatal food poisoning in the Indian Ocean

Jorge Diògene[1*], Laia Reverté[1], Maria Rambla-Alegre[1], Vanessa del Río[1], Pablo de la Iglesia[1], Mònica Campàs[1], Oscar Palacios[2], Cintia Flores[2], Josep Caixach[2], Christian Ralijaona[3], Iony Razanajatovo[4], Agathe Pirog[5], Hélène Magalon[5], Nathalie Arnich[6] and Jean Turquet[7]

Supplementary Figure S1. Assignment test (STRUCTURE software) for shark species identification. Each bar on the *x*-axis represents one individual, and the *y*-axis represents the probability to belong to one or another cluster. The multilocus genotypes of the unidentified shark samples (presumed to be bull shark) were used in assignment with other bull shark individuals[26] and other carcharinid species that successfully amplified the loci used (*Carcharinus obscurus* or *C. plumbeus*). Two clusters were found: the red one corresponds to other shark species and the green one corresponds to bull shark individuals. The unidentified samples clustered with the bull shark cluster.

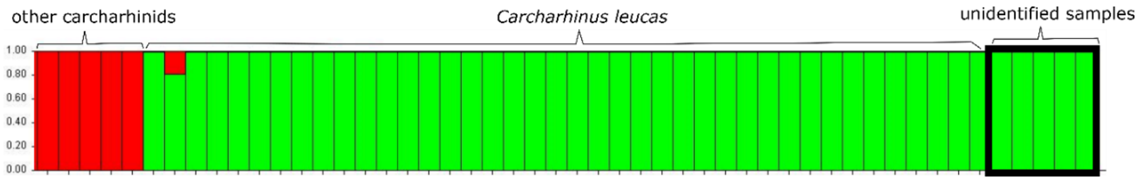

Supplementary Figure S2. Cytotoxicity of P-CTX-1 on Neuro-2a cells with (white dots) and without (black dots) ouabain/veratridine treatment. Error bars represent standard deviation (SD) values for 3 replicates (n=3).

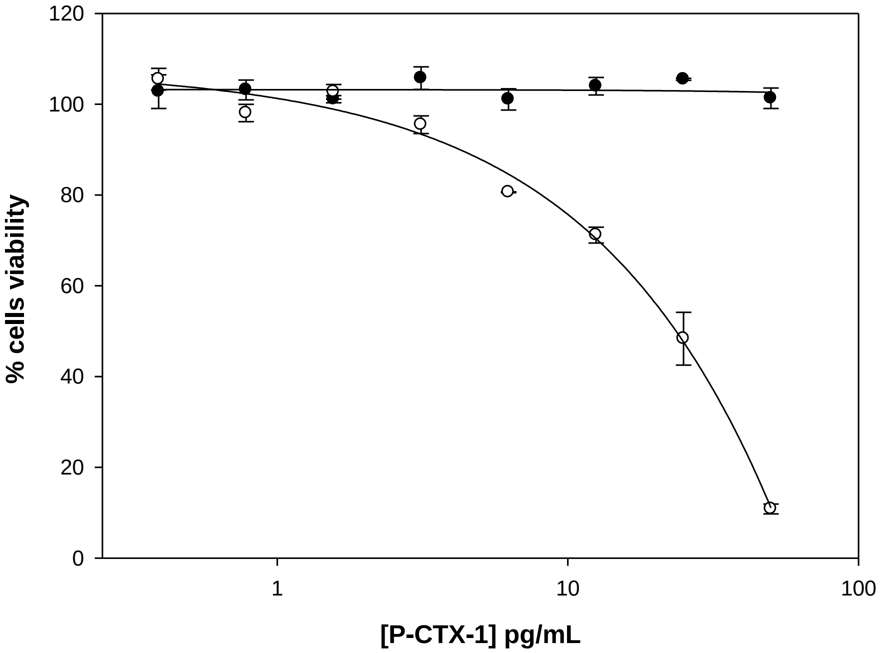

Supplementary Figure S3. Cytotoxicity of fin extract 2 on Neuro-2a cells with (white) and without (black) ouabain/veratridine treatment. In the absence of ouabain/veratridine, a matrix effect leading to cell toxicity was observed at concentrations of 100 mg TE/mL or higher. In the presence of ouabain/veratridine, a CTX-like effect was observed at lower concentrations. (TE: tissue equivalents). Error bars represent standard deviation (SD) values for 3 replicates (n=3).

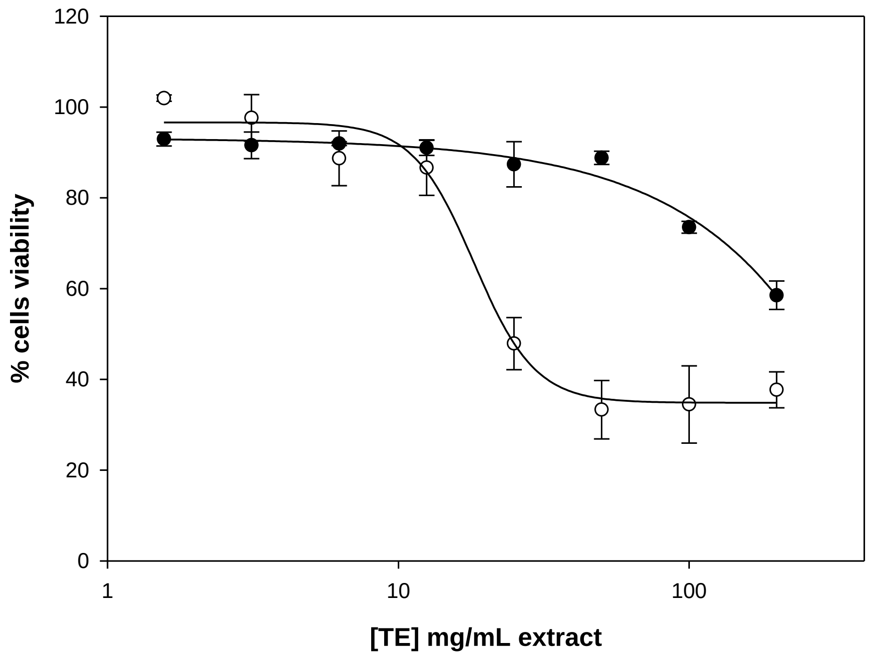

Supplementary Table S1. Multilocus genotypes of the five samples of shark tissue from Madagascar using 20 loci developed for *Carcharhinus leucas* (Cl01 to Cl20[26]), one locus developed for *Galeocerdo cuvier* (Gc01[28]) and one locus developed for *C. limbatus* (Cli106[27]). Alleles are in base pairs and the diploid genotypes are expressed with alleles in 3-digit format and concatenated. NA: no amplification.

Sample Name	Cl01	Cl02	Cl03	Cl04	Cl05	Cl06	Cl07	Cl08	Cl09	Cl10	Cl11
**A565_1: flesh**	116116	143143	106108	124130	137137	148148	149149	148154	111111	127127	232236
**A565_2: stomach**	116116	143143	106108	124130	137137	148148	149149	148154	111111	127127	232236
**A565_3: fin 1**	116116	143143	106108	124130	137137	148148	149149	148154	111111	127127	232236
**A565_4: fin 2**	116116	143143	106108	124130	137137	148148	149149	148154	111111	127127	232236
**A565_5: fin 3**	116116	143143	106108	124130	137137	148148	149149	148154	111111	127127	232236

Sample Name	Cl12	Cl13	Cl14	Cl15	Cl16	Cl17	Cl18	Cl19	Cl20	Gc01	Cli106
**A565_1: flesh**	105105	122124	217217	296296	107109	177177	211211	207215	146155	136136	211211
**A565_2: stomach**	105105	122124	217217	296296	107109	177177	NA	207215	146155	136136	NA
**A565_3: fin 1**	105105	122124	217217	296296	107109	177177	NA	207215	146155	136136	NA
**A565_4: fin 2**	105105	122124	217217	296296	107109	177177	211211	207215	146155	136136	NA
**A565_5: fin 3**	105105	122124	217217	296296	107109	177177	211211	207215	146155	136136	NA
